# “In Sweden you are worthless. In Denmark you get an identity again” – on being perceived and received as a person who uses drugs in different drug policy settings

**DOI:** 10.1186/s12954-024-01035-5

**Published:** 2024-06-17

**Authors:** Julie Holeksa

**Affiliations:** Department of Social Work, Faculty of Health and Society, Malmö University, Nordenskiöldsgatan 1, Malmö, 211 19 Sweden

**Keywords:** People who use drugs, Drug policy, Harm reduction, Stigma

## Abstract

**Background:**

Policies to address substance use differ greatly between settings, where goals may range from zero-tolerance to harm reduction. Different approaches impact formats of care, policing, and even interpersonal interactions, and may play a role in the labelling and stigmatization of people who use drugs (PWUD). Where Sweden has a more restrictive policy, aiming to have a society free from drugs, Denmark has embraced harm reduction principles. The aim of this study was to explore PWUDs’ experiences of interpersonal interactions, policing, and service formats in the two countries.

**Methods:**

The data consists of 17 qualitative semi-structured interviews with Swedish PWUD who have been in both Sweden and Denmark. Recruitment took place at harm reduction sites in both countries, and through snowball sampling.

**Results:**

Participants reflected on how they were perceived by those in public spaces, and received by care systems and personnel. In public settings in Sweden, participants felt they were ignored, rendered invisible, and lost their humanity. In Denmark, they were perceived and acknowledged, valued as people. This was simultaneously linked to being embodied by the availability of differing service offerings and policing practices, which solidified their “right to be out” in public. Reflecting on their reception in the treatment system, strict formatting in Sweden caused participants to feel that an identity was projected upon them, limiting their opportunities or growth of new facets of identity. Care relations in Denmark fostered more opportunity for autonomy and trust.

**Conclusion:**

A zero-tolerance policy and associated public discourses could solidify and universalize stigmatizing categorizations as a central feature of PWUD identity and reception from those around them, exacerbating social exclusion. Conversely, harm reduction-centered policies fostered positive interactions between individuals with care providers, public, and police, which may promote inclusion, empowerment, and wellbeing.

## Introduction

The role of social constructs in producing stigma, identity, social exclusion, and shaping the wellbeing of people who use drugs (PWUD) has long been a consideration of research [1–5]. Numerous works have been devoted to understanding the role of how individuals are received by formal institutions, such as health care and social services, and how informal interactions communicate how individuals are perceived, in response to acts that are defined as “deviant.” “Deviance” is defined as a behaviour which violates social rules. From a social constructionist view, deviance emerges not from the act itself but through societal responses to the act [3]. This perspective underscores that deviance, and consequently stigma, is a result of societal norms and sanctions. For example, the role of criminalization of substance use has been examined in how it perpetuates stigma and reinforces the marginalized position of PWUD [6]. Similarly, negative interpersonal interactions have been shown to informally enforce PWUDs’ stigmatized position [1, 7]. Deviant framing can lead to internalization of stigma, affecting self-perception, and potentially exacerbating negative outcomes associated with drug use, such as increased drug use and overdose risk [8, 9]. It has even been suggested that the primacy and strength of this identity can play a role in the process towards recovery. Specifically, that people who cultivate aspects of their identity that do not involve drug use may be more equipped to cease substance use [5, 10, 11].

However, the precise construction as well as enforcement of social rules around substance use is a complex and subjective process, which can vary significantly between time, place, social group, and individual [3]. This becomes salient when we understand how different countries and their respective drug policies approach substance use. Denmark and Sweden, and their differing drug policies, offer a relevant opportunity to explore how differing approaches to labelling and addressing issues of substance use may influence the experiences and identities of PWUD. In short, while both nations recognize substance use as a social problem, their approaches differ significantly in legal enforcement and care principles. Denmark’s emphasis on harm reduction contrasts with Sweden’s more restrictive approach. This can lead to differences in how people experience they are perceived in public spaces, as well as received by care providing institutions [12, 13].

How, then, do PWUD who have been in both countries experience interpersonal interactions, policing, and service formats in the two settings? The aim of this study is to understand how this group experience that they are met by the system and public alike, within the context of two different drug policy approaches. The study aims to use these experiences to draw conclusions on the greater impact this may have on participants’ identities, sense of inclusion or exclusion, and wellbeing. The case explored in this paper may be used to inform broader discussions when considering different approaches to policy.

## Background

### Swedish /Danish drug policy

As stated, Sweden and Denmark have distinct approaches to issues surrounding substance use. The modern drug policy era began in Sweden in the late 1970’s and 1980’s, when increasing visibility of drug use led to new, highly restrictive measures. The rationale of this approach was largely informed by deterrence theory, in that harsh penalties and negative societal response will deter individuals who may be interested in trying drugs from doing so [14]. In the 1980’s, the goal of “a drug free society” was officially endorsed and personal drug use was criminalized, with a zero-tolerance approach being the officially established strategy [15]. Drug use has been framed as something emerging from outside, as “alien” to Swedish society [16]. The idea that negative attitudes towards drug use may function to limit the normalization and spread of problematic substance use is endorsed in Swedish policy documents from the 1980’s to the present day [17, 18]. Notably, this policy stance in Sweden has been criticized for its departure from evidence-based principles, resulting in a moralistic policy debate [13].

In this context, harm reduction services, which aim to reduce the negative outcomes of drug use without necessarily requiring drug cessation, have developed more slowly in Sweden than in other European countries. While services such as needle and syringe exchange programs (NSP) and opioid substitution therapy (OST) have increasingly embraced harm reduction principles in recent years, full acceptance of harm reduction is perceived as contradicting Sweden’s “zero-tolerance” stance on drug use. Services such as supervised consumption and heroin assisted treatment, for example, have not been implemented. Many services still have relatively high thresholds, and overarching goals of motivating clients to rehabilitation, which have been noted to act as barriers to care for some clients [19–21]. Worthy of note is that there are many regional variations in care options and formats – with Skåne, the southernmost region of Sweden, having a more developed focus on harm reduction as compared to the rest of the country.

Policymakers in Denmark have taken a different approach to substance use than in Sweden. Faced with a parallel emerging drug problem, Danish policymakers saw rising drug use as emerging from within Danish society – as a response to broad social changes which were occurring, and did not want to risk criminalizing and alienating large segments of the population [16, 22]. The Danish government has technically endorsed a zero vision of drug use, however they are clear to remark that harm reduction is not contradictory to this goal and instead is important for those most at risk of harm, as well as the broader community [23]. While fines for drug possession were reinstated in 2004, re-penalization was intended to send a message particularly to recreational and experimental drug users that drug use is unacceptable [22]. Fines however may be waived for people in vulnerable economic situations who have addictions issues, though this policy is not always implemented universally [24]. There are a wider variety of services aimed towards drug use, including services seen to be controversial in Sweden – including supervised consumption rooms, and heroin-assisted treatment. Services are low in threshold and built upon the principles of harm reduction – not requiring the cessation of substance use [22]. The differences in approach are exemplified in figures reflecting access to care – where Denmark has approximately twice as many patients per capita enrolled in OST as Sweden [25]. Similarly to Sweden though, there are regional differences. Specifically, Copenhagen and its Vesterbro neighbourhood are known for an open drug scene and a concentration of harm reduction services [26]. Many, though not all, of the individuals in the study focus their time in this neighbourhood.

Due to their different approaches to criminalization, the countries have differing policing approaches to PWUD. In Sweden, policing and legal policies have progressively become more restrictive towards PWUD – with a previous manifesto that “it should be difficult to be an addict” [27]. Policing in Sweden tends to, though not always [28], be focused on punitive measures for the end user – including occasionally confiscating legally-obtained injection equipment [29]. The police may carry out compulsory drug tests on people suspected of drug use. Conversely, the policing focus in Denmark is more often built on maintaining public order and safety, as opposed to focusing on drug using, especially in neighbourhoods surrounding harm reduction sites [24]. Police in these areas apply a “harm reduction policing” model which is reported to improve, though not entirely abate, policing-related harms [24].

Political circumstances and public attitudes both shape and reflect each other, thus these differing policy approaches may also reflect different attitudes of the public in both countries. Public attitudes are important because they may lead to stigmatizing interpersonal encounters for PWUD and make it more difficult for them to be included in society. Studies of the general public have also found that those with more stigmatizing and negative views towards PWUD are less supportive of harm reduction measures [30]. There are no up-to-date studies which compare public attitudes towards drug use in Sweden and Denmark, however previous studies demonstrate stark differences in views towards cannabis [31] which could reflect current stances.

### Stigma, identity, and PWUD

An important concept in this project is the experience of stigma, and how stigma may be differently enforced and experienced in different settings dependent on institutional goals. Goffman introduced the notion of stigma in social science research, defining it as a (visible or invisible) attribute of one’s identity which is discrediting, and can expose one to being excluded from the dominant society in some way [32]. Identity can be defined not as a static entity but as an ongoing and reciprocal process, involving the interplay between self-perception, societal perceptions, and contextual factors – such as culture, norms, constructs, and laws [33]. Some individuals may be more or less susceptible to stigmatizing experiences and the impact they can have on identity. How PWUD themselves relate and react to the projection of a stigmatized identity on themselves is a highly heterogenous experience. Several studies have shown the different strategies with which people resist, define, or relate to societal narratives about substance use, in relation to their own [5, 34, 35]. McKenna et al. for example show how female methamphetamine users diversely internalized or challenged this identity [5]. Many studies find how people will aim to distinguish different groups, sometimes distancing themselves from the most serious representations [34, 35]. However, it has been suggested that addiction is one of the most stigmatized identities [36]. This form of identity has even been referred to as a “master status” [4], one which has the power to overshadow all other aspects of an identity.

Hatzenbuehler et al. suggest that stigma is a “fundamental cause of population health inequalities,” [37]. This claim is particularly relevant to this study as research has demonstrated that healthcare professionals commonly hold negative perceptions of PWUD, and these negative attitudes consequently are associated with poorer healthcare delivery and treatment outcomes, as well as reduced sense of empowerment amongst PWUD [38]. Perceived and self-stigma also affect treatment and recovery outcomes, including reducing both initial and long-term engagement in care, and self-efficacy [39]. These outcomes can be mediated by, among other factors, harm reduction-based care which limits programmatic controls and/or promotes lack of stigma from staff [39]. Research has shown that internalized identity regarding drug use can have an effect on both physical health and mental wellbeing outcomes [39–43].

Institutional norms and policies have a great influence on the forms of care and people’s disposition towards PWUD [44, 45]. Similar to the application of deviance, Loseke discusses the development of conditions into “social problems,” and suggest that nothing is a social problem until it is constructed and accepted as such [46]. Care systems’ structures, influenced by overarching policy goals, determine who is defined as a client and in what way, service eligibility, and even the legitimacy of certain interventions. In short, it is the system itself which “creates the client” (p. 75) [47]. Loseke describes that organizations designed to address a social problem in fact create and perpetuate a “reality” around that problem [46]. For instance, in settings where abstinence-based education and system goals predominate, service providers may consciously or unconsciously exclude people who have ongoing relapses [48]. Providers with abstinence orientation may not promote harm reduction-based services or goals, as they are not seen as legitimate [44, 49]. Similarly, a policy of criminalization impacts policing practices [50], as seen above in different national policing approaches. The structure of the system influences education, organizational goals, and even attitudes towards PWUD, shaping interactions, where Tempalski & McQuie state that criminalization represents an institutionalization of the exclusion of PWUD [6].

## Methods

The study consists of 17 qualitative, semi-structured interviews, which took place between August 2022 and January 2023. The recruitment setting of the study comprised a border region spanning Sweden and Denmark – referred to as the Öresund region. This region encompasses Eastern Denmark (including the Greater Copenhagen metropolitan area), as well as Sweden’s southernmost region, Skåne. There is no regular border control enacted between the two countries, and there are train, ferry, and road links between, enabling relatively easy mobility. Specific sites of recruitment included a supervised consumption room, as well as an overnight shelter in Copenhagen, Denmark, as well as a needle exchange in Skåne. Additionally, a snowball sampling approach was used, which resulted in two of the participants. To be eligible for inclusion, participants had to be Swedish citizens or residents who had traveled to Denmark and had experiences with accessing harm reduction and other drug-related services there. Importantly, as the people in the study were chosen because they had at some point left Sweden to go to Denmark, the sample is thus comprised of individuals who were often dissatisfied in some way with their circumstances in Sweden. Interviews were conducted in a private office within each organization for those interviewed immediately. Other interviews took place either over the phone, at a private office space, or at cafés/restaurants, depending on the participant’s preference.

The study was approved by the Swedish Ethical Review Authority (Dnr 2019–06509). Informed consent was obtained from individuals to participate and to be audio recorded. Participants were made aware of their right to withdraw at any time or refuse to answer any questions. They were remunerated for their time with a gift card valued at 100DKK/200SEK.The interviews lasted an average of 58 min, with a range of 25 to 111 min, and were transcribed verbatim. The interview guide focused on experiences of accessing care, interpersonal interactions, and policing in the two countries. Notions of stigma, identity, and social inclusion or exclusion most often came up organically by the participant. When not brought up spontaneously, specific questions were asked – for example, how do you feel you are met by people in the general public/treatment professionals/police? How do you feel about the approach to drug use in one or both countries? And how do you feel this impacts you?

The analysis took an abductive approach to thematic analysis [51]. In the first stage, anything of interest with regards to the differences in experiences in the two countries was coded. Then, observing the commonality of ideas of stigma and identity, a coding round focusing directly on elements of stigma, identity, and inclusion or exclusion from society was undertaken. This involved a process of reading and re-reading, making notes, coding data, and re-coding, and developing appropriate thematic categories based on finalized codes.

Of 17 participants, 14 were male, and three were female. Their average age was 41, ranging from 26 to 61. The primary substances of choice were: amphetamines, opioids (primarily heroin and/or methadone), cocaine, and a mixture of heroin/cocaine. Regarding income, four were employed, eight received statutory income support, and three had no formal income source (two did not provide income details). Nine were primarily based in Sweden, eight in Denmark. Of those based in Sweden, two regularly travelled to Denmark, six went occasionally, and one had no such plans. All but one had Swedish citizenship. All had originated from or lived in one of the southernmost three regions of Sweden (Skåne, Blekinge, Småland) at some point in their life, but many had also lived in other more northern areas. Therefore, they represent a diversity of experiences in both Sweden and Denmark The participants in the study were extremely mobile and had been in many different places throughout both countries for longer and shorter periods of time. Seven were currently homeless or had unstable housing, five had never experienced homelessness, and five had experienced homelessness in the past. 16/17 participants had been in both countries within the past 2 years. However, some participants had a decades-long history of mobility between the two countries, and some may be reflecting on more historical experiences.

## Results

Through the coding process, it was seen that participants experiences and reflections revolved around the processes of interactions, perceptions, and reception. These could be categorized into two overarching themes. The first theme being the idea of being perceived in public spaces, relating also to the right to be out. The second theme deals with how participants felt they were being received specifically by the treatment system, and how this related to trust or control.

### Theme I: being perceived and allowed in public spaces

One of the most common themes which was seen in the interviews was the idea of being perceived in public, how daily interactions were shaped by stigmatized constructions of substance use and PWUD, and how this varied between the two countries.

Linking to how drug use and PWUD are viewed and constructed, a common thread discussed by many of the participants was that of how their value as people related to their status as a person who uses drugs. One participant, for example, felt that, “*you don’t get devalued (in Denmark) in the same way as you are in Sweden*.” Similarly, a clear image of this different perception is illustrated by another (as in the titular quote), remarking:

… In Sweden, there you are shit. You are worthless there when you are an addict. Here in Denmark, you get an identity again, you become a person again. A person who is allowed to be seen. Because it was very difficult to go back from Denmark, to Sweden. It was difficult. About your position. When you became a ghost again.

The participant describes that the most difficult part of returning to Sweden from Denmark was becoming a ghost, losing his identity, even losing his humanity. This reflection on feeling as if one is invisible and worthless, speaks to the importance of the basic act of being seen and acknowledged by others. Several individuals remarked on the difference in daily interactions, where in Denmark, people “*actually trying to be nice to you, many times,”* that passersby will “*look you in the eye like anyone else*,” smile at them, and acknowledge their existence. In comparison “*in Sweden people don’t even look at you*,” passersby would commonly change sides of the street, look away. These were behaviours which participants felt to be exhibiting that the public were afraid or simply not able to acknowledge the existence of the participant. Often, participants attributed this behaviour to the popular rhetoric that has been constant in Sweden, that PWUD are criminals, dangerous, a threat to society as a whole. Similarly, another participant put it plainly, reflecting that in Sweden you are treated as “*a criminal*,” whereas in Denmark “*you are a person with a drug problem*.” These quotes speak to the notion of maintaining people’s humanity and value through policy and discourse – how a deviant and a problem is constructed and ascribed, and what influence this has on daily interpersonal interactions and even a sense of personal value.

A related phenomenon was recounted as participants’ sense of their right to be in public, expressed by one participant as the freedom “*to be seen, to be out*.” This was felt to be confirmed by the interpersonal interactions conveyed above. The above-expressed fear of PWUD, projected by people changing sides of the street in Sweden, in turn then led to people reporting resistance strategies such as proactively concealing themselves – reporting “*I hide myself*,” – to avoid having to face these sorts of interactions. They reported feeling that these interactions led to them being pushed, or, pushing themselves, concealing themselves, into the margins of society both literally and metaphorically.

Participants’ stories also suggest how the countries’ drug policies informed policing, which influenced their feelings of safety, freedom, participation, and the ability to move freely in public spaces. This freedom to take place in public was described as being symbolically reinforced by the availability of services, and the form of policing practices. The same participant who reported hiding himself, remarked “*it’s different there, a different feeling, they think differently. They have space, you didn’t have to search, worry, think about space, where to sit and smoke, because it’s hard to find…. It was much better. It was what I needed the most and you could get it from there.”* This participant refers to the availability of services like supervised consumption (not existing in Sweden), and the lack of punitive policing practices. In Denmark, there were spaces where they could use drugs, developed for them, with them in mind. The zone around the consumption room, where policing actions in general focus on public safety rather than individual drug possession and use, also generated these sorts of sentiments. One participant recounting an encounter in a medium-sized Danish city when the police approached him while he was injecting publicly while the consumption room was closed for cleaning. He became scared and nervous, but rather than arresting him as he expected, the police instead re-directed passersby. They told him that they would protect him while he took his fix, something which he reflected on appreciatively with strong emotion. In Sweden, participants reported feeling chased out of public spaces, where, “*you are woken up if you sit and sleep or nod off. You are woken up by the police or security guards. There in Denmark, they check if you’re okay, don’t bother you. [They ask] ‘Are you okay?”* Where drug crime pursuit is much more punitive, many felt in a constant state of worrying, one participant noting in his small hometown, he couldn’t go “*one meter without being stopped by the police … I was being marked there*.” These accounts describe the impact of differing drug policies and social environments on individuals’ feelings of acceptance and inclusion as part of society. These experiences affirm or deny peoples’ rights to be in a space, and can also lead to trust or mistrust with authorities – something which is explored in the next theme.

### Theme II: being received in care

The second theme relates to how participants felt they were received, particularly by the format of services and attitudes of service providers, and how this may have an impact on trust, self-image, and even in some cases, participants’ substance use trajectory. This is intrinsically linked to how policy goals shape which sorts of services are offered, and in what way.

Several participants discussed that within the Swedish treatment system, an identity was projected upon them (or that they perceived was being projected), influencing the way they were treated. One participant, remarking on his experiences in OST, stated that he did not “*recognize myself in that image, as, like a fucking addict… that you aren’t trustworthy, that you manipulate, you lie, that’s how a drug user is, that is the image*” which he reflected was “*incredibly harmful, that is the worst that I know.*” This reflection underscores a dissonance between the ascribed identity and self-perception, highlighting the emotional toll of being misjudged and feeling discriminated against based on how a PWUD is constructed in the treatment setting. This experience suggests an issue where the individuals felt trapped by a “spoiled identity,” perceiving it as an inescapable label that significantly affected their treatment and overall wellbeing.

Participants also reflected on differing approaches to relapse in the two countries’ treatment systems. One participant expressed that she had a brief relapse while in OST in Denmark, and “*when I was honest, they didn’t punish me, but (treatment) continued as normal*.” She compared this to similar experiences in Sweden, when a relapse was something to hide, and when it came to the attention of staff, “*it feels like you are punished instead… you have been shamed if you should relapse, you start lying to them instead*.” Here she relates not only to being penalized with reduced doses, but also a sense of shame being ascribed or projected, experiencing that the reaction of the system in general generated a sense of discrediting, in letting oneself down, and necessitating a controlling response. Similarly, another participant noted that if she told her Danish case worker or treatment providers that she had relapsed, or was having strong drug cravings, “*it’s not gonna have negative consequences for me, she’s actually gonna try to help me. And I know that, and that helps me trust the system*.” Both participants feared the negative consequences of honesty, which in their experiences had been punished in Sweden. This led to people reporting concealing relapses and drug urges when on treatment, rather than having earlier intervention to find the underlying cause of the issue. They also had to go through a process of learning that the approach in Denmark would yield different results. Being in a place of trust was noted to give participants a feeling of empowerment.

One participant also compared the experience of going to in-patient rehabilitation in Sweden and in Denmark. Where in Sweden, she noted, “*they check your bag, they check your everything*,” in Denmark instead they expected her to leave any drugs or paraphernalia voluntarily with staff upon intake, her response to that being:

*and I was like you’re gonna trust me? Like what? And that was so superb. That did so much for me, because I was like, well they put this trust in me, I don’t want to let them down, you know. And that meant so much to me.*

This shift in perspective was transformative for a participant who eventually relocated to Denmark, where she felt a newfound sense of hope and identity:

*All of a sudden I have dreams that I believe in … when I lived in Sweden, I couldn’t see it. … always being ‘one of the addicts’ and within that category. But today I don’t feel like I’m in that category. Today I feel like I’m a fighter, I’m ready… (In Sweden), it feels like they think we’re liars, … But here (in Denmark) they show that they trust you… here they say ‘yes we think you can do it’*

In the quote, the participant discusses how the attitude of service providers, feeling that they believed in her and trusted her, were key factors in leading to a change of her identity, her aspirations for her future, and conceptualizations of what she could achieve. Her sense of self shifted, and her trust in herself developed in response to these interactions. It prompted her to see herself not only as someone who uses drugs, but as someone capable of cultivating other facets of her identity. Some of this may be relate to a process of maturation, and the influence of a change of environment in her move from Sweden to Denmark. However, she specifically reflected on how it related to being met by those around her involved in her care. She reflects that the support she has received has had a profound impact on her outlook, inspiring hope, and new ambitions.

How controlling care formats reinforce stigma was contrasted with the ability to have autonomy over care which led to a sense of empowerment. Control or autonomy was also linked to a sense of humanity, which was also reflected in one participant’s comments about care:

*that they (treatment staff in OST) gave me a lot of freedom, that was only positive. Like, otherwise you only encounter stigma or limitations as a drug user….It was quite, liberating and nice, and a little more humane as well.*

Because of travel distance between his OST provider and his home, this participant was allowed to have take-home doses once a month. This was despite being open with his provider about occasional side use of illicit drugs. The participant reflects that normally he is controlled by care limitations as defined by the system due to his status as a “drug user,” meaning care providers do not believe that he can be trusted to have autonomy over his own care. He discusses the humanity of a treatment format which is less controlling and acknowledges the person behind the label, and the importance of being treated as an individual with inherent value beyond his drug use. Freedom in this setting facilitated a sense of liberation from the constraints typically imposed on PWUD.

Participants generally described interactions in Sweden as more negative, but there was variation between different interventions. The personnel at low threshold interventions such as NSP were described as helpful and understanding, with one participant remarking, “*the people who work there are great. When I need to seek, or receive care, I prefer to go to them…I would rather go there than to my normal doctor*,” however “*It feels like it has stagnated; it’s just an institution that remains the same, with the same content since [the Nineties]*” Here, the interactions are framed as positive, but the formatting of those services still was restrictive and dated in comparison to the possibilities in Denmark. In many cases, due to the limited hours, restrictions on equipment distributions, and required blood borne virus testing, this led to people avoiding this service and going directly to Denmark instead.

## Discussion

The study reveals contrasts in experiences of societal attitudes and institutional practices towards PWUD who have been in both a restrictive setting, Sweden, as well as one with more embrace of harm reduction, Denmark. The data demonstrate the multiple layers in which stigma can dynamically interfere with wellbeing. Previous research has noted how stigma impacts care formats and legitimacy of services [48, 52, 53], care outcomes and access [38, 42, 54, 55], mental health [56], and overdose [9]. Importantly, the results also show how this stigma may differentiate in different settings. As described by Bjerge et al., Denmark has constructed substance use as emanating from within society – a reflection of societal problems – which may afford individuals a more inclusive and less stigmatizing environment [16]. This is evidenced by participants’ accounts of greater empowerment and humanity in their interactions with service providers and the public. Whereas in Sweden, substance use has been framed as external to its society, and something which is endeavored to be eliminated completely. This may promote a more punitive and exclusionary stance that reinforces the “master status” [4] of a stigmatized identity and undermine trust in systemic support. These differential constructions have tangible effects on PWUD’s daily lives, self-perception, access to care and treatment relations, influencing their perceived value, social inclusion, and views of the future.

The experiences reported by participants pertain to aspects of the system itself, and to their beliefs about how they are perceived by others, what Kaufman & Johnson describe as “reflected appraisals” [57]. Anderson in fact argues that *perceptions* of marginalization heavily influence wellbeing, identity development, and harms [58]. This is similar to how Savonen et al. discuss “situational identity negotiation,” exploring how PWUD position themselves within dominating social representations of addiction [34]. Participants in the study were therefore not merely passive recipients, and are instead involved in the process of interpreting and negotiating their experiences. Many participants in this study actively rejected the stigmatizing labels, indicating that they did not identify with how they were being treated. However, despite not always identifying *with* the stigmatized identity, they often felt they were being identified as it by others, which had clear influence on their lived experience. One resistance strategy was even to physically distance themselves from the environments that devalued them – a topic explored elsewhere [59]. The results therefore also highlight the context-bound nature of stigmatized identities, which can be modified or shed in different settings, suggesting a fluidity of these statuses.

These narratives shed light on the broader implications of how PWUD are managed within care systems. Research highlights the role of empowering environments, social acceptance, and support, in fostering individual wellbeing, engagement in healthcare, and pathways to recovery [53, 60, 61]. Empowerment is characterized by giving individuals a sense of control and agency over their own care, and more broadly their lives [61], and is a concept which has been officially endorsed and weaved into Danish welfare policy documents (though dilemmas do exist in practice) [53]. Empowerment can be linked with the concept of “self-trust,” defined by McLeod & Sherwin as confidence in one’s values, decisions, and autonomy [62]. While oppression leads to internalizing societal beliefs of inferiority, undermining self-trust, supportive social environments can instead cultivate self-trust [62]. This emphasizes the need for healthcare systems to foster patient autonomy [62]. Trust and its importance for care outcomes for PWUD has been explored in works by Lago et al. and Trealoar et al., among many others [63, 64]. Conversely, systems imbued with control (as are common in care relating to PWUD) have been linked with negative outcomes such as disengagement, disempowerment, dissatisfaction, and exclusion of the most vulnerable from treatment [35, 39, 65–67]. Harris & McElrath discuss the controls of OST programs, such as supervised doses and frequent urine drug screening, as reinforcing a drug using “master status,” [65]. These notions can be reflected in the results of this study, where an emphasis on punitive measures and mistrust may lead to a cycle of deceit and hiding. Conversely, when individuals are given autonomy over their recovery, it may foster a sense of agency, self-reliance, and empowerment.

On the other hand, full client autonomy and harm reduction has been critiqued in relation to care of PWUD, a prominent concern being that it promotes a sense of fatalism among care providers and clients, lacking enough of a motivational drive for PWUD to recover fully or reach a goal of drug freedom in the long term, as well as places a burden of responsibility on clients [68]. Addiction-related care in Sweden is seen to be highly ambitious, with a greater focus on traditional goals of rehabilitation and recovery, which could be beneficial for some. Importantly, a report on 11 different Swedish OST programs reveals overall positive levels of satisfaction (64%) [69]. The views of these clients may not be reflected in this study.

It is largely agreed upon that it is not just the form or even quality of treatment options, but also internal processes which drive perceptions of future, and ability for people to recover from substance use. A variety of literature highlights the pivotal role of developing alternative facets of identity, such as a “recovery identity”, enabling individuals to move away from the label of “drug user” as a key factor in desisting from problematic substance use [11]. For example, the notion of self-narrative momentum, described by McConnell, relates to how people view the future and their prospects of hope [70]. These works could be related to the current study with regards to, for example, participants reporting new positive conceptions of their prospects for the future, and new aspects of their identity emerging. While critics such as Fomiatti et al. suggest that “recovery identity” is overly individualizing [71], this paper instead places the impetus not only on individual, but on societal factors, to change. Mechanisms influencing stigma, empowerment, and exclusion or inclusion, could facilitate identity transformation.

Public views of PWUD and interactions may reinforce the exclusion of this group. Stigmatizing messaging in general is associated with desire for social distance [72], and stigmatized language towards PWUD has been found to increase negative perceptions of this group [73]. A study of community pharmacy users in Scotland noted avoidance of PWUD who used harm reduction services there [74], similar sorts of interactions which could be mirrored by the results of this study. In some cases, stigmatizing public interactions led to participants avoiding going out in public, which resulted in a self-fulfilling prophecy of exclusion for some participants. Several studies have also explored the public’s views of substance use, notions of responsibility, and willingness to pay for treatment or offer harm reduction [30, 75]. These findings reflect some of the perceptions of “worthiness” or “value” of PWUD seen in this study’s results.

The idea of belonging in a space also raises pertinent questions about the dynamics of inclusion and exclusion, and the influence of local contexts. A study mapping life conditions and risks for PWUD in Copenhagen and Malmö found that those in Sweden felt more chased by police [76]. This was qualitatively reflected in this study, that Danish police were willing to protect the rights of participants, despite their use of illicit drugs. Swedish PWUD in the [76] study also got more help from each other, rather than official sources, with protection, as well as for resources such as food, sleeping places, drugs, and more. Limited access to low threshold services and fear of police therefore may result in increased involvement in the drug economy, exacerbating marginalization and complicating efforts toward social integration [77]. Further exploring the notion of belonging, Kammersgaard investigated PWUD-related policy and discourse by local stakeholders in two Danish cities, Aarhus and Copenhagen [78]. The author found that in Aarhus, PWUD were represented as being “out of place” or infringing on the perceived rights of “normal people”. In Copenhagen, they were seen as being “in place” [78]. As most (though not all) in the current study had spent the majority of their time in Copenhagen, some of the results relating to “right to be out” may be a more localized phenomenon to the Copenhagen neighbourhood that many participants spent their time in. These findings also highlight how public policy and messaging plays a role in creating belonging, worthiness, and public acceptance of this group.

The construction of a certain type of stigmatized group, and how this influences care formats, interpersonal interactions, and the internalization of this narrative, is a complex social process influenced by many factors. Although Sweden has slowly been embracing more harm reduction principles in care, it has simultaneously been strictly maintaining the overarching goal and policy narrative of a “society free from drugs.” The messaging that this type of policy engenders involves a level of inherent stigma towards drug use, and certain controls in care formats. The results of the study suggest that for some, a zero-tolerance policy framework could solidify and universalize the categorization of a “drug user” as a central element of their self-perception and reception from those around them, potentially exacerbating their societal exclusion. Conversely, Denmark’s national policies fostered more possibilities for positive interactions between individuals and care providers, as well as with the wider public, which are factors which may promote inclusion, empowerment, wellbeing, and the development of a multifaceted identity. This underscores the importance of understanding the complex dynamics between policy, societal attitudes, and the shaping of individual experiences.

### Limitations

The study has a number of limitations. Firstly, the sample size is relatively small and may not capture all of the complexities or the range of experiences of the phenomenon being studied. The experiences of stigma discussed here are may not be generalizable to other groups, nor are they necessarily representative of the experiences of all people who have experiences of both the Danish and Swedish system. Secondly, the participants’ demographic characteristics were largely similar, with limited representation of groups such as women. This is particularly important as women for example are noted to face additional and different layers of stigma in their substance use [5]. This limits the exploration of intersectionality in experiences of stigma and exclusion. Finally, some of the experiences may be outdated and could be based on past experiences with restrictive programs in Sweden, which may have deterred more recent engagement with services which have evolved in the latest years.

## Data Availability

The datasets generated during and/or analyzed during the current study are not publicly available due to privacy concerns.
